# Rash and Nephrotic Syndrome in a Patient with Rheumatoid Arthritis

**DOI:** 10.34067/KID.0000000000000102

**Published:** 2023-06-29

**Authors:** Zein Alabdin Hannouneh, Serena Bagnasco, Mohamad Hanouneh

**Affiliations:** 1 Al Andalus University Faculty of Medicine, Al Andalus University for Medical Sciences, Tartus, Syria; 2 Department of Pathology, Johns Hopkins Medical Institutions, Baltimore, Maryland; 3 Division of Nephrology, Department of Medicine, Johns Hopkins University School of Medicine, Baltimore, Maryland; 4 Nephrology Center of Maryland, Baltimore, Maryland

**Keywords:** drug nephrotoxicity, glomerular disease, nephrotic syndrome, renal biopsy, SLE

## Abstract

**Figure:**
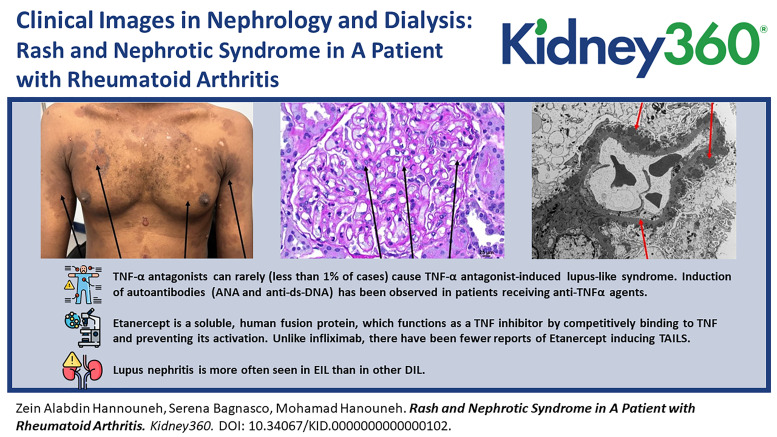

## Case Description

**Figure 1:**
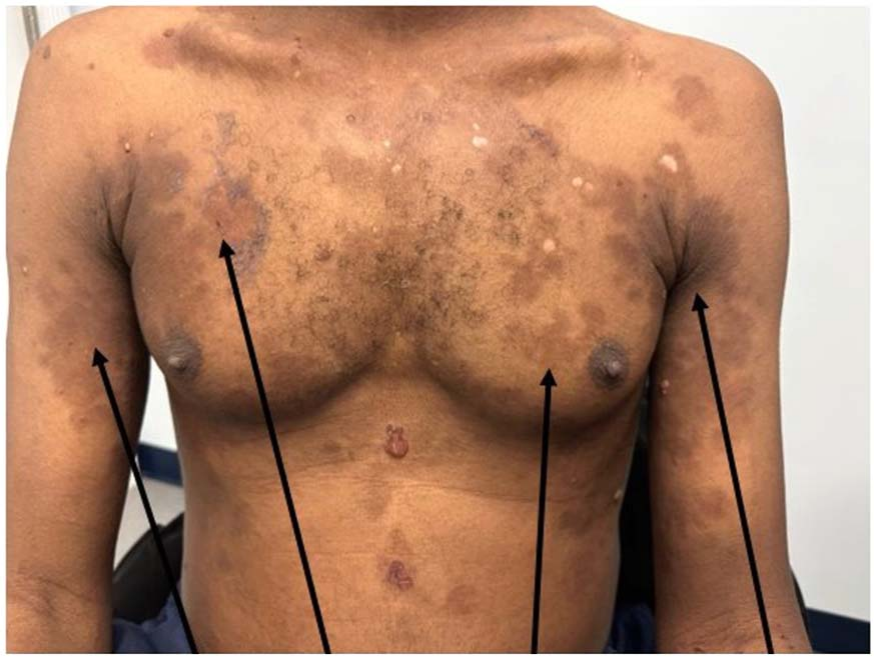
Discoid-like rash over patient's chest and upper extremities.

**Figure 2:**
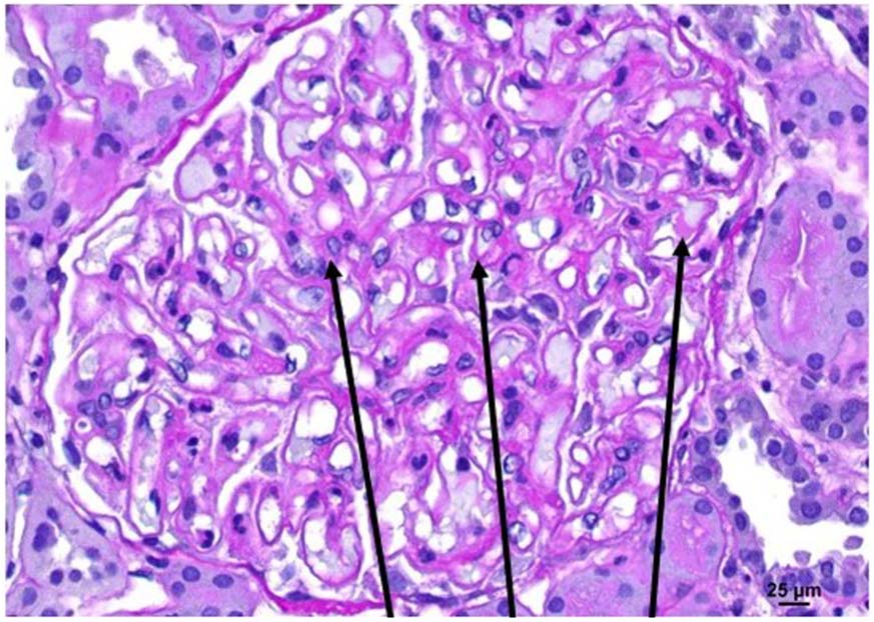
**Kidney biopsy, light microscopy-PAS stain showing diffuse thickening of the glomerular capillary walls, and mesangial expansion.** PAS, periodic acid–Schiff.

**Figure 3:**
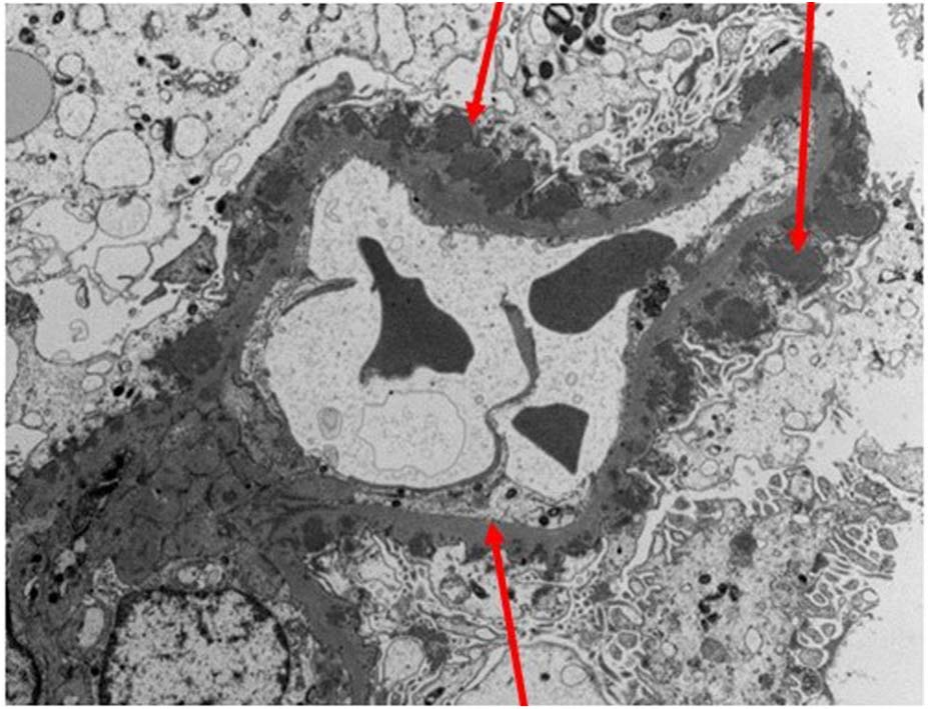
Kidney biopsy, electron microscopy, showing diffuse thickening of the glomerular basement membrane with numerous subepithelial and intramembranous electron dense deposits, along with diffuse foot process effacement.

A 41-year-old man with a history of rheumatoid arthritis presented to the emergency department with lower extremity edema and rash. He had been started on weekly etanercept infusions (50 mg) 3 months prior. His vitals were normal on arrival, and cardiopulmonary, abdominal, and neurologic examinations were all unremarkable. He had +2 lower extremity edema and a discoid rash over his chest and upper extremities (Figure 1). Initial laboratory values were as follows: white blood cell count 6.96×10^9^/L, hemoglobin 11.4 g/dl, platelets 202×10^9^/L, sodium 136 mmol/L, potassium 3.6 mmol/L, chloride 105 mmol/L, bicarbonate 23 mmol/L, BUN 17 mg/dl, serum creatinine 0.82 mg/dl, calcium 7.7 mg/dl, albumin 1.4 g/dl, aspartate aminotransferase 78 U/L, and alanine aminotransferase 32 U/L. Urinalysis was remarkable for +3 proteinuria and hematuria with 11–25 red blood cells per high-power field. Urine protein/creatinine was 6.2 g/g. Patient underwent kidney biopsy, with light microscopy showing diffuse thickening of the glomerular capillary walls and mesangial expansion (Figure 2 periodic acid–Schiff stain). The interstitium was free of inflammation or fibrosis. The tubules revealed red blood cells in the tubular lumen. Immunofluorescence demonstrated granular staining in the capillary walls, mesangium, and the tubular basement membrane for IgG, IgA, kappa, lambda, C3, IgM, and C1q. Electron microscopy revealed diffuse thickening of the glomerular basement membrane with numerous subepithelial and intramembranous electron dense deposits, along with diffuse foot process effacement (Figure 3). Serology workup later revealed the following: positive antinuclear antibodies (ANA) 1:2560, positive anti–ds-DNA 1:320, and positive anti-histone IgG >7 units. The patient was diagnosed with etanercept-induced membranous lupus nephritis. The etanercept was stopped, and the patient was started on mycophenolate mofetil 1000 mg twice daily and prednisone 40 mg daily, with plan to taper both medications.

## Discussion

Drug-induced lupus (DIL) is the occurrence of a lupus-like syndrome after exposure to certain medications. TNF-*α* antagonists have been commonly used to manage several autoimmune conditions and can rarely (<1% of cases) cause TNF-*α* antagonist-induced lupus-like syndrome (TAILS).^1^ Etanercept is a soluble, human fusion protein, which functions as a TNF inhibitor by competitively binding to TNF and preventing its activation. Unlike infliximab, there are fewer reports of etanercept inducing TAILS.^2^ Etanercept-induced lupus (EIL) mostly affects women, and the onset of symptoms takes a mean of 4.4 months from the start of medication.^1^ Symptoms of EIL usually include SLE cutaneous features such as malar rash, discoid rash, and photosensitivity. Other systemic symptoms include fever, weight loss, arthritis, serositis, and myositis. Although neurological and kidney involvement is rare, class III and IV lupus nephritis and less commonly class V are more often seen in EIL than in other DIL.^3,4^ Induction of autoantibodies (ANA and anti–ds-DNA) has been observed in patients receiving anti-TNF*α* agents.^1,3^ The presumed mechanism is induction of apoptosis promoting antibody production against uncleared antigenic nucleosomes.^5^ The diagnosis of TAILS is based on history and clinical features. Kidney biopsy will be helpful in the setting of kidney involvement. In almost all cases, the disease abates after cessation of the causative drug. However, immunosuppressive agents are required in complicated cases including organ involvement such as lupus nephritis.^3^

## Teaching Points

- TNF-*α* antagonists can rarely (<1% of cases) cause TAILS. Induction of autoantibodies (ANA and anti–ds-DNA) has been observed in patients receiving anti-TNF*α* agents.
- Etanercept is a soluble, human fusion protein, which functions as a TNF inhibitor by competitively binding to TNF and preventing its activation. Unlike infliximab, there have been fewer reports of etanercept inducing TAILS.
- Lupus nephritis is more often seen in EIL than in other DIL.
